# Data on fluoride concentration level in villages of Asara (Alborz, Iran) and daily fluoride intake based on drinking water consumption

**DOI:** 10.1016/j.dib.2016.09.050

**Published:** 2016-10-05

**Authors:** Giti Akhavan, Sina Dobaradaran, Jaleh Mohajeri Borazjani

**Affiliations:** a Department of Environmental Engineering Bushehr branch, Islamic Azad University, Bushehr, Iran; b The Persian Gulf Marine Biotechnology Research Center, Bushehr University of Medical Sciences, Bushehr, Iran; c Department of Environmental Health Engineering, Faculty of Health, Bushehr University of Medical Sciences, Bushehr, Iran; d Systems Environmental Health, Oil, Gas and Energy Research Center, Bushehr University of Medical Sciences, Bushehr, Iran; e Department of Fisheries and Natural Resources, Bushehr Branch, Islamic Azad University, Bushehr, Iran

**Keywords:** Asara, Fluoride, Groundwater, Spring

## Abstract

In the present data article, fluoride concentration levels of drinking water (with spring or groundwater sources) in 10 villages of Asara area located in Alborz province were determined by the standard SPADNS method using a spectrophotometer (DR/2000 Spectrophotometer, USA). Daily fluoride intakes were also calculated based on daily drinking water consumption. The fluoride content were compared with EPA and WHO guidelines for drinking water.

**Specifications Table**

**Table:** 

*Subject area*	*Chemistry*
*More specific subject area*	*Daily fluoride intake*
*Type of data*	*Table and figure*
*How data was acquired*	*Spectrophotometer (DR/2000 Spectrophotometer, USA)*
*Data format*	*Raw, analyzed*
*Experimental factors*	*All water samples in polyethylene bottles were stored in a dark place at room temperature until the fluoride analysis.*
*Experimental features*	*Determine the concentration levels of fluoride*
*Data source location*	*Asara area, Alborz, Iran*
*Data accessibility*	*Data are included in this article*

**Value of the data**

• Data can be used as a base-line data for concentration levels of the fluoride in spring and groundwater.
• The data shown here will be informative for health policy makers by assigning interception actions against adverse health effects of fluoride with considering fluoride intake by drinking water and food.
• Data shown here may serve as benchmarks for other groups working in the field of water, food, and toxicology to compute organic and inorganic daily intakes by drinking water as well as food consumption.

## 1 Data

In the Asara area of Alborz province the concentration levels of fluoride in their drinking water sources (spring and groundwater) ranged from 0.1–3.19 mg/L (Mean 0.763). As seen in Table 1, it shows that the average daily intakes of fluoride based on 2 liter daily drinking water consumption [1] reached 1.52 with a range of 1.02–2.7 mg/day. As shown in Table 1, the mean concentration levels of fluoride in drinking water of all villages were below than the EPA, and WHO drinking water guidelines.

## 2 Experimental design, materials and methods

### 2.1 Study area description

**Fig. 1:**
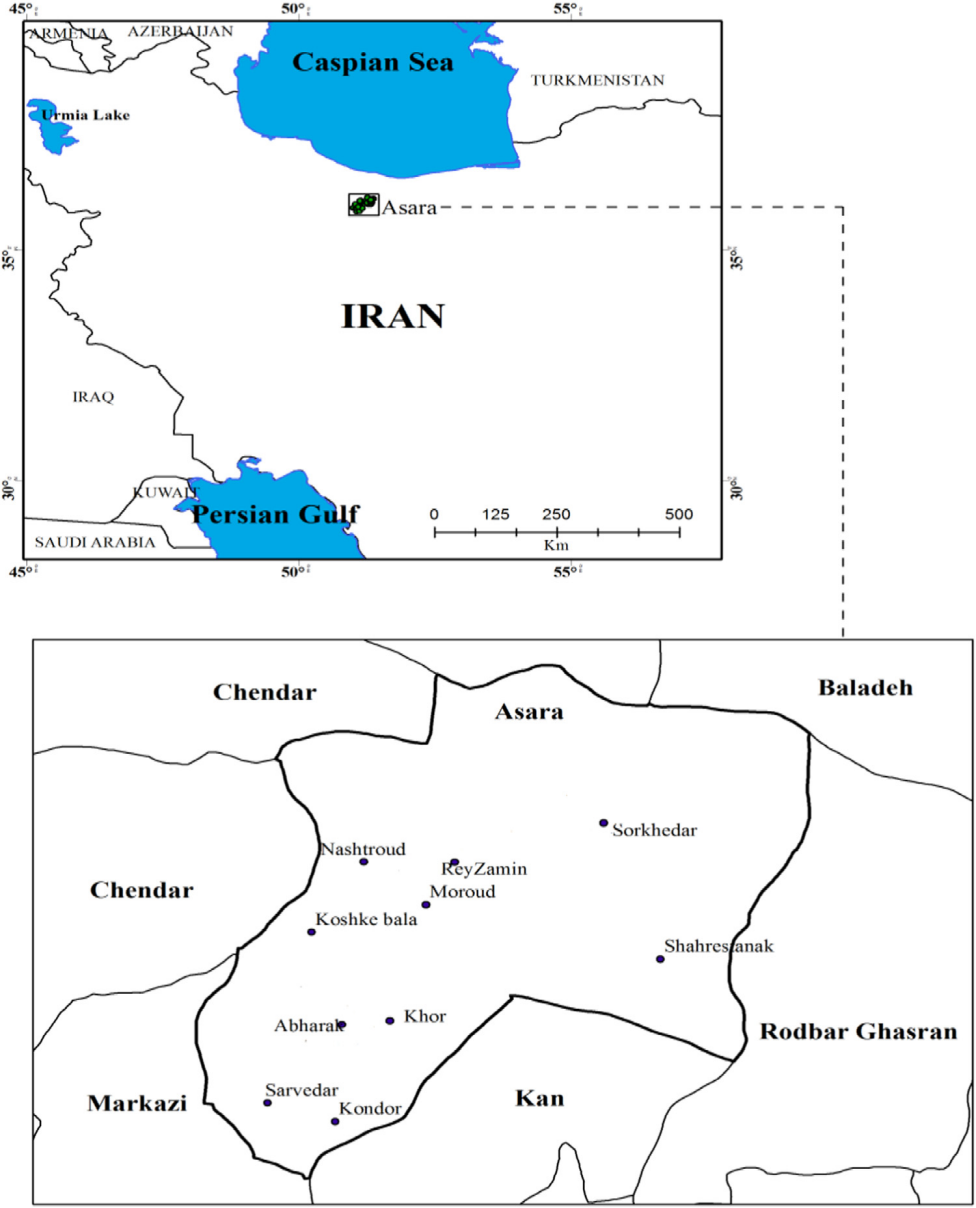
The map and locations of sampling villages.

Ten villages of Asara (Alborz, Iran) were selected as sampling points including Sorkhedar, Sarvedar, Khor, Kondor, Moroud, Nashtroud, Abharak, Shahrestanak, Rey Zamin, and Koshke Bala (Fig. 1).

### 2.2 Sample collection and analytical procedures

Sixty samples were collected from 10 villages (6 samples from each village) of the Asara area of Alborz province. Water samples were collected by using 200 mL polyethylene bottles that were washed three times with deionized water; prior to collecting each sample, then bottles were labeled with the sample number and location for identification. After transferring to the laboratory, all samples were stored in a dark place at room temperature in polyethylene containers until the fluoride analysis was made by the standard SPADNS method [2], [3], [4], [5], [6], [7], [8], [9], [10], [11], [12], [13], [14], [15], [16] using a Spectrophotometer (DR/2000 Spectrophotometer, USA). The concentration levels of fluoride in waters were compared with EPA and WHO guidelines for drinking water. Eventually daily fluoride intakes were estimated based on 2 l daily drinking water consumption and concentration levels of fluoride in waters.

## Figures and Tables

**Table 1:** Mean concentration levels of fluoride (mg/l) in drinking water of the Asara area of Alborz province, comparison with EPA and WHO guidelines for drinking water, and daily fluoride intakes.

**Village**	**Source**	**Fluoride concentration (mg/L)**		**Daily intake (mg/day)**
		**Range**	**Mean**	
Sorkhedar	Spring	0.19–1.53	0.73±0.51	1.46
Sarvedar	Spring	0.15–1.48	0.7±0.52	1.4
Khor	Spring	0.14–1.54	0.55±0.54	1.1
Kondor	Spring	0.15–1.28	0.54±0.44	1.08
Moroud	Spring	0.16–1.09	0.51±0.33	1.02
Nashtroud	Spring	0.15–1.28	0.55±0.5	1.1
Abharak	Spring	0.1–3.19	0.73±1.2	1.46
Shahrestanak	Groundwater	0.2–3.11	0.99±1.06	1.98
Rey Zamin	Groundwater	0.75–2.68	1.35±0.71	2.7
Koshke Bala	Spring	0.23–2.13	0.98±0.71	1.96
Minimum value	–	0.1	0.51	1.02
Maximum value	–	3.19	1.35	2.7
Average value	–	–	0.763	1.52
EPA standard	–	2	–	–
WHO standard	–	1.5	–	–

*Based on 2 liter daily drinking water consumption and concentration levels of fluoride in drinking waters.
